# Suppressing subordinate reproduction provides benefits to dominants in cooperative societies of meerkats

**DOI:** 10.1038/ncomms5499

**Published:** 2014-07-22

**Authors:** M. B. V. Bell, M. A. Cant, C. Borgeaud, N. Thavarajah, J. Samson, T. H. Clutton-Brock

**Affiliations:** 1 Department of Zoology, Large Animal Research Group, University of Cambridge, Downing Street, Cambridge CB2 3EJ, UK; 2 Centre for Ecology and Conservation, University of Exeter in Cornwall, Penryn, Cornwall TR10 9EZ, UK; 3 Kalahari Meerkat Project, Kuruman River Reserve, PO. Box 64, Northern Cape 8467, South Africa; 4 Mammal Research Institute, University of Pretoria, Pretoria 0002, South Africa; 5 Present address: Institute for Evolutionary Biology, University of Edinburgh, Ashworth Laboratories, Kings Buildings, West Mains Road, Edinburgh EH9 3JT, UK; 6 Present address: Institute of Biology, University of Neuchâtel, 2000 Neuchâtel, Switzerland; 7 Present address: Institute of Evolutionary Biology and Environmental Studies, University of Zurich, Winterthurerstrasse 190, CH-8057 Zurich, Switzerland

## Abstract

In many animal societies, a small proportion of dominant females monopolize reproduction by actively suppressing subordinates. Theory assumes that this is because subordinate reproduction depresses the fitness of dominants, yet the effect of subordinate reproduction on dominant behaviour and reproductive success has never been directly assessed. Here, we describe the consequences of experimentally preventing subordinate breeding in 12 groups of wild meerkats (*Suricata suricatta*) for three breeding attempts, using contraceptive injections. When subordinates are prevented from breeding, dominants are less aggressive towards subordinates and evict them less often, leading to a higher ratio of helpers to dependent pups, and increased provisioning of the dominant’s pups by subordinate females. When subordinate breeding is suppressed, dominants also show improved foraging efficiency, gain more weight during pregnancy and produce heavier pups, which grow faster. These results confirm the benefits of suppression to dominants, and help explain the evolution of singular breeding in vertebrate societies.

## Abstract (web summary)

In hierarchical animal societies, dominant females suppress breeding by subordinate females. Here, the authors experimentally suppress breeding by subordinate female meerkats and observe that dominants give birth to heavier pups which grow faster, suggesting this behaviour improves fitness of dominants.

## Graphical abstract

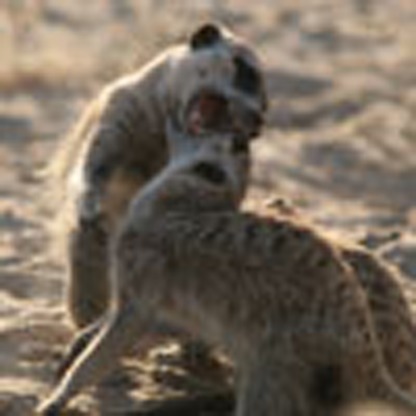

Understanding the evolutionary paradox of eusociality requires that we explain the origins of extreme reproductive inequality within stable societies^1^,^2^,^3^,^4^. Research has been dominated by theoretical models, often with limited empirical support^5^,^6^,^7^,^8^,^9^. A principal assumption has been that group-living females compete over the resources required to breed, and that dominants benefit from the reproductive inactivity of subordinates^2^,^6^,^10^. Conflict is expected to be particularly acute in cooperative breeders, where non-breeding subordinates usually contribute to offspring care. Here, subordinates themselves may represent a resource, which is depleted when they attempt to breed and start investing in their own offspring, rather than contributing to cooperative offspring care. This is thought to explain why dominant females in cooperative breeders commonly suppress subordinate reproduction by targeted aggression, temporary eviction or infanticide^11^,^12^,^13^.

A secondary assumption in an important subset of models (the ‘tug-of-war’ models and their derivatives) is that attempting to exert control over the distribution of reproduction is itself costly, reducing the total reproductive output^5^,^6^,^7^,^8^,^14^. Selection to minimize the cost of fighting may explain why dominant efforts at suppression are not inevitable, instead appearing to be sensitive to variation in the payoffs of interfering with subordinate breeding: attacks are targeted at subordinates who are most likely to breed^15^; are restricted to periods when resource competition peaks and the offspring of dominants may be at a competitive disadvantage^15^,^16^; or are avoided entirely when the subordinate retaliation is likely to be effective^17^.

Evidence for both assumptions is correlational and open to interpretation. Evidence for negative effects of subordinate reproduction on dominants is limited to observations that subordinate breeding is sometimes associated with reductions in aspects of dominant female reproductive success, including care received by offspring^18^, offspring weight at independence^15^ and offspring survival^13^,^16^,^19^. It is unclear that this is causal, and may be due to age or condition related declines in dominant condition or capacity to prevent subordinate breeding. Evidence for the cost of attempting to alter the distribution of reproduction by interfering with the reproduction of other individuals is restricted to a few observations of declines in the quality of offspring subsequently produced by dominants, the quality of care they receive or their probability of survival^20^,^21^. Again, it is difficult to rule out correlations with other social or ecological variables (food availability in particular).

## Results

### Experimental protocol

We experimentally tested the assumptions of reproductive skew theory using injections of the contraceptive hormone Depo-provera (medroxyprogesterone acetate, at 7.5 mg kg^−1^) to prevent subordinate female reproduction in our long term study population of wild, habituated meerkats at the Kuruman River Reserve, South Africa (26°58'S, 21°49'E). Meerkats are cooperative mongooses, living in groups of 3–50, where reproduction is monopolized by a dominant pair^15^ and all subordinates contribute to offspring care by babysitting and provisioning pups. Dominant females breed two to four times per year, and pups remain in their natal burrow for 3 weeks before starting to forage with the group, when they are provisioned by adults until c. 3 months. Groups contain several (1–12) adult subordinate females, who also attempt to breed. Subordinate breeding attempts usually trigger intense aggression by dominant females, culminating in temporary eviction from the group, or infanticide of subordinate pups^11^,^15^,^16^.

In year 1, in six treated groups, all subordinate females over 180 days old (*n*=35 females) were injected with contraceptive, and in six control groups, all subordinate females (*n*=38) were injected with an equivalent volume of saline solution. Initial injections were administered during the first week of July 2009, the middle of the dry season, when the probability of pregnancy is at its lowest. Two further injections were administered at 90-day intervals (October 2009 and January 2010), giving a total treated duration of 9 months (July 2009–March 2011 inclusive). During this period, 14 of the control females (at least one in each of the 6 Control groups, range 1–5 per group) were detected to have conceived at least once (range of conceptions 1–3 per female), while no treated females were detected to have conceived. In year 2, the protocol repeated, starting in July 2010 and ending in March 2011, with control groups from year 1 now receiving Depo-provera (*n*=72 females), and treated groups from year 1 now receiving saline (*n*=38 females). During this period, 14 of the control females were detected to have conceived: two Control groups showed no subordinate conceptions, while all the remaining groups had at least three subordinates who conceived at least once (range 3–4 conceiving females, with conceptions ranging from 1–3 per females). In contrast, no treated females were detected to have conceived during this period. Overall, the experiment affected 59 dominant female breeding attempts (33 control and 26 treated).

### Dominant female behaviour

**Figure 1:**
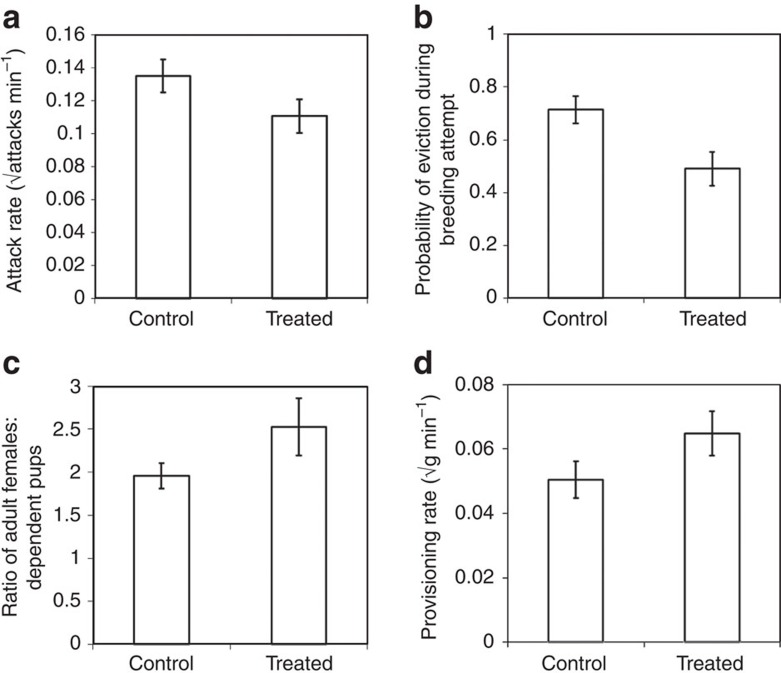
The effect of experimental suppression of subordinate female reproduction on: (**a**) the rate at which dominant females attack subordinates (analysis conducted on 1,952 focal watches of 12 dominant females); (**b**) the probability that a subordinate female was evicted during a breeding attempt (analysis conducted on 128 subordinate females, present at 59 breeding attempts (33 control and 26 treated) by 12 dominant females; (**c**) the ratio of adult females to dependent pups during the period of peak pup provisioning (20 to 40 days after birth; analysis conducted on 59 breeding attempts (33 control and 26 treated) born to 12 dominant females); and (**d**) provisioning rates by subordinate females (mass of food per minute, square root transformed; analysis was conducted on 1,050 focal watches, of 72 subordinate females). Means ±s.e. Sample sizes may be less than complete experimental sample because it was not always possible to observe specific animals within target time windows.

Throughout the experiment, we visited the groups at least twice per week, to collect behavioural data, record group composition and life history events, and weigh animals (who have been trained to step onto portable electronic lab scales). Every week, we conducted at least two 30-min focal watches on each dominant female (to give a total of 1,067 h of observations on 12 females), recording every instance of aggression directed towards subordinate females. Dominant females attacked treated subordinates at lower rates (linear mixed model (LMM), F_1,1951_=8.74, *P*=0.003; Fig. 1a.; Supplementary Table 1). We also recorded the total amount of time when at least one subordinate female was within 2 m of the dominant, finding that dominants were more tolerant of the presence of treated subordinates (LMM F_1,1951_=8.03, *P*=0.005; Supplementary Table 2). Similar focal observations on the two largest subordinate females currently present in each group (960 h of focal observations on 99 females) recorded the outcome of each foraging attempt. These revealed that treated females were less likely to be interrupted by the dominant female during a foraging bout (generalized linear mixed model (GLMM), F_1,1812_=11.13, *P*<0.001; Supplementary Table 3).

To determine whether the reduction in aggression affected subordinate eviction, we investigated the probability of eviction during the dominant female’s gestation for 128 subordinate females, present at 59 breeding attempts (33 control and 26 treated). Treated females were less likely to be evicted by the dominant female during her gestation (GLMM F_1,127_=8.38, *P*=0.004; Fig. 1b; Supplementary Table 4). The analysis also revealed that larger (closer in size to the dominant) and older subordinates were more likely to be evicted (GLMM, size: F_1,127_=6.51, *P*=0.012; age: F_1,127_=4.82, *P*=0.029; Supplementary Table 4), confirming previous findings that dominants target those most likely to attempt to breed themselves^15^. It is unclear how dominants detected the suppressed reproductive state of subordinates: it is likely that olfactory cues played a role^22^, and there were some behavioural changes in subordinates (for instance subordinates were less submissive: LMM F_1,1951_=6.77, *P*=0.009; Supplementary Table 5);. However, there were no overall changes in activity patterns or affliative behaviour: no change in movement (proportion of time observed spent actively moving; LMM F_1,1812_=0.14, *P*=0.71); no change in vigilance behaviour (time observed standing on hind legs, scanning for predators; LMM F_1,1812_=0.03, *P*=0.87); no change in allogrooming behaviour with other subordinate females (time spent grooming with another subordinate female; LMM F_1,1812_=1.18, *P*=0.28).

### Helper:pup ratio

Under natural conditions, evicted females frequently fail to return, either because they die, or because they establish new groups with unrelated males^23^. Eviction may therefore reduce the number of helpers present in a group, with negative effects on pup development^24^. Reducing the eviction rates may therefore increase the number of helpers present during pup provisioning, which we assessed by calculating the average number of adult females (>360 days old) present between 20 and 40 days after birth (the period during which pups are primarily dependent on provisioning by adults). We restricted the analysis to females because (i) they contribute more than males to pup provisioning^25^; and (ii) the number of males fluctuates due to temporary absences while prospecting for mating opportunities in other groups^26^. The ratio of female helpers to pups was greater in treated groups (LMM F_1,58_=5.89, *P*=0.019; Fig. 1c; Supplementary Table 6), which is likely to have substantial positive effects on subsequent pup development.

### Dominant female weight gain and pup emergence weight

**Figure 2:**
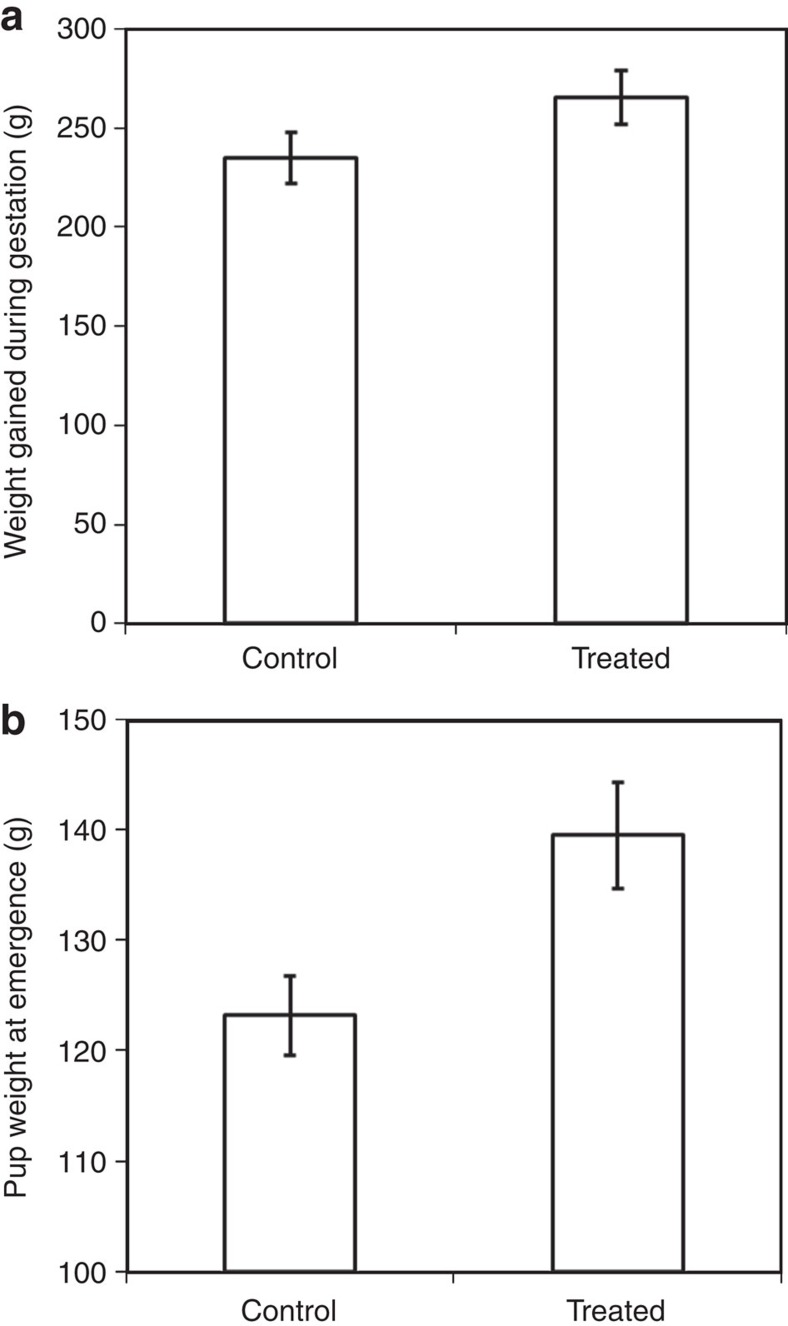
The effect of experimental suppression of subordinate female reproduction on: (**a**) weight gain by dominant females during pregnancy (analysis conducted on 12 females over 54 pregnancies (23 treated and 31 control)).; and (**b**) pup weight at emergence (analysis conducted on 215 pups (128 treated, 87 control) from 51 litters (22 treated and 29 control) born to 12 dominant females. Means ±s.e. Sample sizes may be less than complete experimental sample because it was not always possible to weigh animals within target time windows.

To test whether dominants benefited from reduced aggression toward subordinates, we analysed dominant foraging efficiency, since foraging may have been less interrupted. Our focal watches recorded time spent foraging and prey biomass captured, and revealed that dominant females in treated groups captured more food per minute (interaction between dominance and treatment, F_1,3764_=4.63, *P*=0.032; Supplementary Table 7). This increase in foraging efficiency, coupled with the reduced effort invested in evicting subordinates, meant that dominant females in treated groups gained more weight during pregnancy (LMM F_1,53_=5.62, *P*=0.022; Fig. 2a; Supplementary Table 8), and pups born to dominant females in Treated groups were heavier when they first emerged from their burrows (LMM F_1,214_=6.46, *P*=0.021; Fig. 2b; Supplementary Table 9; *n*=128 Treated, 87 control pups). The strength of the effect is emphasized by the fact that pup weight was also affected by the number of subordinate females allolactating (F_1,214_=24.59, *P*<0.001), and there were fewer allolactating females in treated groups (LMM F_1,58_=7.59, *P*=0.008; Supplementary Table 10). The positive effect of additional allolactators implies that successful reproduction by subordinates carries additional costs to dominants, since surviving subordinate pups would detract from the milk available to dominant pups.

### Subordinate female helping effort

Helpers in cooperative breeders are thought to face a trade-off between investment in cooperative offspring care and investment in their own reproduction^27^,^28^. Therefore, we expected subordinate females in treated groups to increase their helping effort, both because they were unable to invest in their own reproduction, and because they were less subjected to the metabolic cost of eviction. We confirmed this by analysing pup provisioning rates, finding that subordinate females in treated groups provided more food (LMM F_1,1049_=5.80, *P*=0.016; Fig. 1d; Supplementary Table 11), but with no change in dominant provisioning rate (LMM F_1,1165_=0.48, *P*=0.49; Supplementary Table 12). In contrast, provisioning rates declined in Control groups when females (both dominant and subordinate) were themselves pregnant (LMM F_1,1471_=6.02, *P*=0.014; Supplementary Table 13).

### Pup growth rate

**Figure 3:**
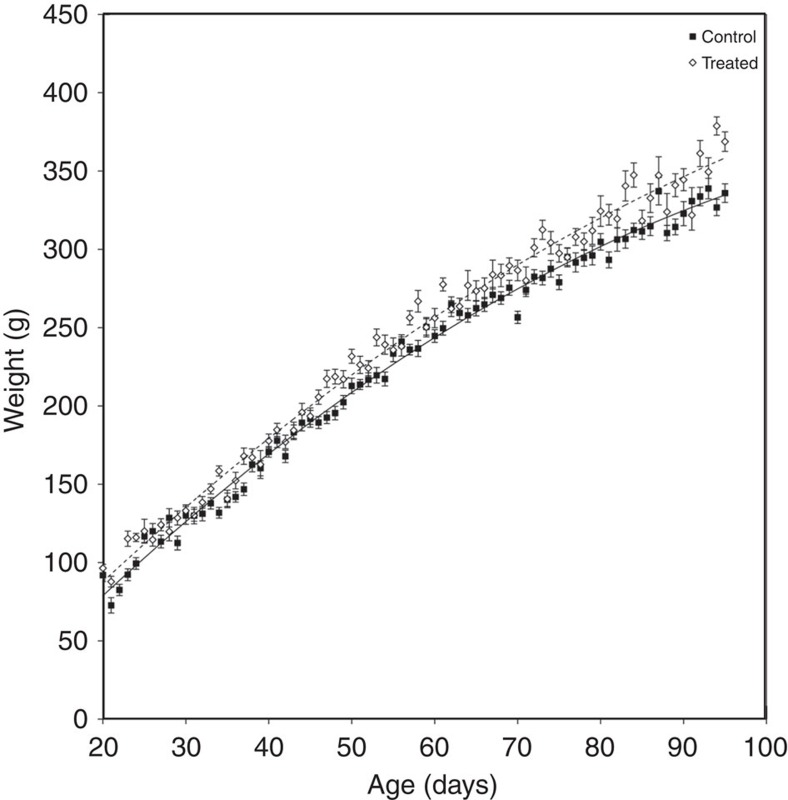
The effect of experimental suppression of subordinate female reproduction on dominant pup growth between emergence and nutritional independence. Analysis conducted on 7,620 morning weights, taken from 241 pups (141 males, 100 females) from 59 litters (26 Treated and 33 Control), born to 12 dominant females. Data points are average weights for all pups weighed at that age (±s.e.), lines are predicted means generated by the model.

Pups in treated groups started life heavier, but were also in groups with more helpers, many of whom were feeding at higher rates, so we expected them to show elevated growth rates after emergence. Analysis of pup morning weight revealed that pups in treated groups grew faster between emergence and 95 days (LMM interaction between treatment and age F_1,7619_=4.35, *P*=0.03; interaction between treatment and age^2^ F_1,7619_=30.66, *P*<0.001; Fig. 3; Supplementary Table 14). Pup weight at emergence and independence is likely to have profound long term effects on pup fitness: size at emergence determines competitive ability in early life^18^; experimental feeding to increase pup weight increases survival^24^; and size at adulthood affects probability of attaining dominance, dominance tenure and reproductive success^29^.

## Discussion

Understanding the origins of extreme reproductive inequality within societies requires that we measure the individual consequences of variation in the extent to which reproduction is shared within a society. Our results confirm the principal theoretical assumption that dominants are selected to suppress subordinate breeding because it reduces dominant fitness^1^,^2^,^5^,^6^,^7^,^8^,^9^, and we demonstrate that helpers themselves represent a resource which is depleted when they attempt to breed. However, we also demonstrate that controlling subordinate breeding imposes substantial costs on dominants^7^,^8^,^20^, as indicated by increased dominant foraging success, gestational weight gain and pup emergence weight in treated groups.

Given the costs of subordinate reproduction, and the costs of preventing it, we should expect dominants in cooperatively breeding species to develop low cost mechanisms for restricting subordinate reproduction. Rather than direct attack, dominants may simply reduce opportunities for subordinate reproduction, for instance by denying access to unrelated members of the opposite sex. We should also expect dominants to deploy tactics which reduce the cost of conflict, for instance by exaggerating power asymmetries, via strategic resource allocation in early life.

Our results also suggest why plural breeding is rare in cooperative vertebrates: dominants are only likely to tolerate subordinate reproduction when it has little effect on dominant reproductive success, which is only likely where social structure limits direct competition between offspring. This may explain why banded mongooses, a closely related social mongoose, are one of the few cooperative vertebrates where multiple females commonly breed together^13^: direct competition between pups is limited because pups are cared for by a single helper who does not provision other pups^30^.

## Methods

### Study site and study population

The experiments were conducted on our long term study population of wild, habituated meerkats at the Kuruman River Reserve, South Africa (26°58’S, 21°49’E), between June 2009 and June 2011. The study site experiences two distinct seasons: a hot-wet season (October–April) and a cold-dry season (May–September), though there is considerable interannual variation in rainfall^31^. Full details regarding the study site and population can be found elsewhere^31^,^32^. Rainfall was measured daily (in mm) using a standard rain gauge on site. On days for which rainfall data were missing (<1%), we imputed rainfall data from a remote-sensing data set provided by the NASA GES DISC (Goddard Earth Sciences Data and Information Services Center) Giovanni online data system^33^.

All animals in the population were tagged with PIT tags for permanent identification, and could be identified during behavioural observation by small dye marks on their fur. Animals were habituated to close observation (from 1 m), and were trained to step onto a portable electronic weighing balance before the morning foraging session, allowing regular measures of body mass to be collected. All procedures conducted during the course of this study were approved by Northern Cape Conservation and the Ethical Committee of the University of Pretoria.

### Capture protocol

Target females were captured by hand, placed in a cloth bag, anaesthetized with isoflurane, injected, placed in a box to recover and released within 15 min In total, 138 females were captured 362 times, with no measurable negative effects. Not all females present in a group at the start of a treatment period were captured during subsequent capture sessions due to death or dispersal.

### Life history data

Each group was visited on at least 2 days per week for the duration of the experiment, usually for 3–5 h in the morning (from dawn) and 2–3 h in the evening (until dark). We visited groups daily when birth approached, so most birth dates were known to within 1 day. We recorded a full group composition at each visit.

Dominant individuals were identified by their behaviour towards other group members^4^,^15^,^16^. They scent-marked more frequently than subordinates, and frequently asserted their dominance over other animals by anal marking, chin rubbing or physical attack. Subordinates responded to dominance assertions by adopting a submissive posture, often accompanied by a characteristic vocalization.

Pregnancy was detected from abdominal swelling and associated weight gain from around 3 weeks after conception. Birth was identified by sudden overnight weight loss of >100 g, obvious change in body shape and the onset of babysitting. Conception date was retrospectively estimated as occurring 70 days before birth. A breeding attempt was included in the analysis if the first injection (treatment or control) of the year in that group occurred at least 60 days before the birth of that litter (that is, gestation primarily occurred in the presence of experimentally suppressed subordinates). Litters that were born between 1 and 59 days after the first injection were excluded from analysis (*n*=3). Allolactators were identified by distended nipples, flattened and moist fur or observed suckling by pups.

We included as potential candidates for eviction all subordinate females who were present in a group 70 days before the birth of a litter, and survived until at least 1 day after birth (either in the group or outside the group). We analysed the probability that they were evicted at least once during the gestation of that litter. Evictions were identified when a female spent at least one night separated from its group, and had been observed receiving aggression from the dominant during the 2 days before separation.

We calculated the average number of adult females (>360 days old) present in each group between 20 and 40 days after birth (the period during which pups are primarily dependent on provisioning by adults). We restricted the analysis to females because (i) they contribute more than males to pup provisioning^25^; and (ii) the number of males fluctuates widely due to temporary absences while prospecting for mating opportunities in other groups^26^.

### Weights data

For pup emergence weight, the first weight collected for each pup was used, with a cutoff at 45 days (in some cases morning weight; in some cases a capture weight; average age at first weight 30.9 days, range 20–45 days). For dominant female conception weight, we calculated the average morning weight between 80 and 70 days before birth. For birth weight, we calculated the average morning weight between 1 and 5 days before birth. Weight gain during gestation was calculated as the difference between birth weight and conception weight (*n*=54, five pregnancies not included because either conception weight or birth weight were not recorded).

### Behavioural data

Every week, we conducted 1 h of focal observations on each dominant female (two 30-min sessions on the same day, at least 30 min apart), to give 1,067 h of focal observations on 12 females; and we collected 30-min of focal observations on the two largest subordinate females currently present in each group (to give 960 h of focal observations on 99 females). We recorded the total time spent foraging (actively digging for food or scratching at the surface) and the outcome of each foraging attempt (success, failure or interrupted by another individual). We assigned successful foraging bouts a prey item size category, which was converted into a prey mass estimate based on previously sampled food items of that size (see ref. 32 for details), and recorded whether or not it was fed to a pup. We recorded the duration, outcome and partner identity of all social interactions (initiated and received aggression, submissive behaviour and allogrooming). For dominant females, we recorded the total amount of time when at least one subordinate female was within 2 m; for subordinate females, we recorded the total amount of time spent within 2 m of the dominant female. Behaviours were recorded using a bespoke programme running on Psion II (LZ64) personal organizers.

### Statistical analysis

Statistical analyses were conducted using Genstat, version 15 (VSN International 2013). Where multifactorial analyses involved repeated sampling of individuals, litters or groups, mixed models were used. These are similar to general(ized) linear models but allow both fixed and random terms to be included^33^. All models involving linear data with a normal distribution were analysed using LMMs, while binomially distributed data were analysed using GLMMs with a binomial error structure and a logit link function. In four models (Model 1, dominant female aggression; Model 8, subordinate female pup provisioning; Model 9, dominant female pup provisioning; Model 10, dominant and subordinate pup provisioning in control groups), the response variable was square root transformed to normalize the data. In all mixed models, random terms were retained, unless the variance component was found to be zero (and hence their removal did not influence the findings reported). In each model, all potential explanatory terms were entered and dropped sequentially until only those terms that explained significant variation remained. The significance of a term was derived by dropping it from the final model (if it was part of the final model), or adding it to the final model and then dropping it (if it was not part of the final model)^34^,^35^. We tested all two-way interactions, but only present those explaining significant variation. All statistical tests were two tailed. Unless otherwise stated, means are quoted ±1 s.e. We present the effect sizes of all significant terms—these are parameter estimates from the models and can be interpreted as the change in *y* per unit change in *x*. For categorical variables, such as sex, one level of the factor is set at 0, and the effect is relative to that factor level.

## Author contributions

T.H.C.-B., M.B.V.B. and M.A.C. planned the experiments, which were conducted by C.B., J.S., N.T. and other members of the meerkat team; M.B.V.B. analysed the data and drafted the results; M.B.V.B. and T.H.C.B. wrote the paper.

## Additional information

**How to cite this article:** Bell, M. B. V. *et al*. Suppressing subordinate reproduction provides benefits to dominants in cooperative societies of meerkats. *Nat. Commun.* 5:4499 doi: 10.1038/ncomms5499 (2014).

## Supplementary Material

Supplementary Information Supplementary Tables 1-14
