# Supplementary material for: Suppressing subordinate reproduction provides benefits to dominants in cooperative societies of meerkats
Source: Nat Commun. 2014 Jul 22;5:4499. doi: 10.1038/ncomms5499 (PMC4109011; doi:10.1038/ncomms5499)
Supplement: Supplementary Information — Supplementary Tables 1-14 [file ncomms5499-s1.pdf]

Supplementary Table 1: LMM of variables influencing rates of aggression initiated by dominant females. Analysis conducted on 1952 focal watches on 12 dominant females. Response variable square root transformed to normalise the data. Identity of dominant female fitted as random term.

**Explanatory terms**	**F**	**df**	***p***
% time spent within 2m of at least one subordinate female*	55.13	1	<0.001
Treatment year (1 or 2)	51.04	1	<0.001
Dependent pups in group (yes/no)	19.58	1	<0.001
Treatment	8.74	1	0.003
Reproductive status (non-breeding/lactating/pregnant)	5.29	2	0.005
Weight (g)	4.03	1	0.045
Age (days)	3.90	1	0.061
Rainfall over previous 60 days (mm)	0.95	1	0.331
Treatment order	0.48	1	0.49

**minimal model**	**effect size**	**s.e.**
Constant	0.088	0.012
% time spent within 2m of at least one subordinate female	0.20	0.027
Treatment year (1 or 2)	0.066	0.0092
Dependent pups in group (yes)	-0.036	0.0082
Treatment (Treated)	-0.019	0.0066
Reproductive status: Lactating	0.0	
Pregnant	0.0024	0.0085
Non-breeding	0.027	0.0097
Weight (g)	0.000081	0.000040

**Random Terms**	**Estimated variance**		**s.e.**
Dominant ID	0.00095	0.00051	

*Result is qualitatively unchanged if number of adult subordinate females is included instead.

Supplementary Table 2: LMM of variables influencing proportion of time observed with at least one subordinate female within 2m of the Dominant female. Analysis conducted on 1952 focal watches on 12 dominant females. Identity of dominant female fitted as random term.

**Explanatory terms**	**F**	**df**	***p***
Dependent pups in group (yes/no)	15.86	1	<0.001
Treatment year (1 or 2)	8.56	1	0.004
Treatment	8.03	1	0.005
Number of subordinate females in group	7.84	1	0.007
Weight (g)	5.16	1	0.023
Rainfall over previous 60 days (mm)	4.22	1	0.040
Reproductive status (non-breeding/lactating/pregnant)	2.10	2	0.122
Treatment order	0.01	1	0.92
Age (days)	0.0	1	0.97

**minimal model**	**effect size**	**s.e.**
Constant	0.11	0.0078
Dependent pups in group (yes)	-0.024	0.0061
Treatment year (2)	0.019	0.0065
Treatment (Treated)	0.016	0.0055
Number of subordinate females in group	0.0092	0.0033
Weight (g)	-0.000075	0.000033
Rainfall over previous 60 days (mm)	0.00073	0.000036

**Random Terms**	**Estimated variance**	**s.e.**
Dominant ID	0.00051	0.0027

Supplementary Table 3: GLMM of variables influencing proportion of subordinate foraging bouts that were interrupted by the Dominant female. Analysis conducted on 1813 focal watches on 99 subordinate females. Identity of Dominant females and Subordinate female fitted as random terms.

**Explanatory terms**	**F**	**df**	***p***
Foraging efficiency (grams found per min of active foraging)	134.58	1	<0.001
Treatment year (1 or 2)	48.10	1	<0.001
Rainfall over previous 60 days (mm)	21.73		<0.001
Treatment	11.13	1	<0.001
Dominant female reproductive status (non-breeding/lactating/pregnant).	3.01	2	0.05
Treatment order	3.66	1	0.073
Weight (g)	2.68	1	0.10
% time spent within 2m of dominant female	2.63	1	0.11
Dependent pups in group (yes/no)	1.54	1	0.21
Total time spent foraging	1.32	1	0.25
Age (days)	0.73	1	0.39
Weight difference between subordinate and dominant (g)	0.0	1	0.95

**minimal model**	**effect size**	**s.e.**
Constant	-5.59	0.16
Foraging efficiency (grams found per min of active foraging)	1.49	0.13
Treatment year (1 or 2)	0.73	0.10
Rainfall over previous 60 days (mm)	-0.0063	0.0014
Treatment (Treated)	-0.36	0.11
Dominant reproductive status: Lactating	0.0	
Pregnant	0.39	0.13
Non-breeding	0.25	0.12

**Random Terms**	**Estimated variance**	**s.e.**
Dominant ID	0.012	0.019
Individual ID	0.015	0.031

Supplementary Table 4: GLMM of variables influencing probability that a subordinate female was evicted during a breeding attempt. Analysis conducted on 128 subordinate females, present at 59 breeding attempts (33 Control and 26 Treated) by 12 dominant females. Breeding attempt, dominant female identity and subordinate female identity were fitted as random terms.

**Explanatory terms**	**F**	**df**	***p***
Treatment	8.38	1	0.004
Weight difference between subordinate and dominant (g)	6.51	1	0.012
Subordinate age (days)	4.82	1	0.029
Number of subordinate females in group	2.89	1	0.094
Dominant age (days)	1.49	1	0.24
Treatment year	1.25	1	0.27
Total rainfall during gestation period	0.02	1	0.89
Treatment order	0.82	1	0.38
Number of adults of both sexes in group	0.24	1	0.63

**minimal model**	**effect size**	**s.e.**
Constant	0.80	0.23
Treatment (Treated)	-0.79	0.27
Weight difference between subordinate and dominant (g)	-0.0036	0.0014
Age (days)	0.0012	0.00055

**Random Terms**	**Estimated variance**	**s.e.**
Dominant ID	0.060	0.016
Individual ID	0.32	0.99
Breeding attempt	0.47	0.22

Supplementary Table 5: LMM of variables influencing the rate at which dominant females received submissive behaviour from subordinate females. Analysis conducted on 1952 focal watches on 12 dominant females. Identity of dominant female fitted as random term.

**Explanatory terms**	**F**	**df**	***p***
Dependent pups in group (yes/no)	44.91	1	<0.001
Weight (g)	38.39	1	<0.001
Age (days)	22.68	1	<0.001
Number of subordinate females in group	21.56	1	<0.001
Treatment	6.77	1	0.009
Reproductive status (non-breeding/lactating/pregnant)	5.59	2	0.004
Rainfall over previous 60 days (mm)	2.29	1	0.13
Treatment order	1.49	1	0.22
Treatment year (1 or 2)	0.12	1	0.73

**minimal model**	**effect size**	**s.e.**
Constant	0.19	0.015
Dependent pups in group (yes)	-0.057	0.0086
Weight (g)	0.00022	0.000036
Age (days)	0.000088	0.000019
Number of subordinate females in group	0.023	0.0050
Treatment (Treated)	-0.018	0.0069
Reproductive status: Lactating	0.0	
Pregnant	0.0019	0.0093
Non-breeding	-0.03	0.013

**Random Terms**	**Estimated variance**	**s.e.**
Dominant ID	0.0013	0.00070

Supplementary Table 6: LMM of variables influencing the ratio of adult females to dependent pups (number of females >360 days old/litter size at emergence)*. Analysis conducted on 59 breeding attempts (33 Control and 26 Treated) born to 12 dominant females. Identity of mother fitted as a random term.

**Explanatory terms**	**F**	**df**	***p***
Treatment	5.89	1	0.019
Rainfall during gestation (mm)	4.41	1	0.041
Mother's weight at conception (g)	0.41	1	0.53
Mother's age at birth (days)	0.27	1	0.61
Treatment Year	0.08	1	0.78
Treatment Order	0.91	1	0.35

**minimal model**	**effect size**	**s.e.**
Constant	2.02	0.15
Treatment (Treated)	0.41	0.17
Rainfall during gestation (mm)	0.0019	0.00091

**Random Terms**	**Estimated variance**	**s.e.**
Mother ID	0.14	0.098

*Result is qualitatively unchanged if ratio of *all* adult helpers (male and female) to pups is analysed as the response variable (Treatment F=4.18, p=0.046, effect = 0.59 ±0.29).

Supplementary Table 7: LMM of variables influencing the foraging efficiency (grams of food found per minute of active foraging) of both Dominant and Subordinate females. Analysis was conducted on 3765 focal watches (1952 watches on 12 Dominant females; 1813 watches on 99 Subordinate females). Identity of Group and focal female fitted as a random terms. Response variable square root transformed.

**Explanatory terms**	**F**	**df**	***p***
Rainfall over previous 60 days (mm)	186.94		<0.001
Age (days)	17.33	1	<0.001
Dominance status (Dominant or Subordinate)	12.07	1	0.002
Treatment Year	6.66	1	0.01
Number of adults present in group	7.57	1	0.014
Treatment	5.67	1	0.017
Dominance status * Treatment	4.63	1	0.032
Pups present in group (yes/no)	2.71	1	0.10
Number of adult females present in group	2.63	1	0.12
Treatment order	1.74	1	0.20
Weight (g)	1.21	1	0.27
Reproductive status (non-breeding/pregnant/lactating)	1.01	1	0.60

**minimal model**	**effect size**	**s.e.**
Constant	0.51	0.010
Rainfall over previous 60 days (mm)	0.00066	0.000048
Age (days)	0.000040	0.0000078
Dominance status (Dominant)	0.038	0.011
Treatment Year (2)	0.024	0.0092
Number of adults present in group	-0.0056	0.0021
Treatment (Treated)	0.019	0.0079
Dominance status * Treatment (Dominant * Treated)	0.035	0.016

**Random Terms**	**Estimated variance**	**s.e.**
Group ID	0.0019	0.00024

Individual ID	0.00064	0.00030

Supplementary Table 8: LMM of variables influencing weight gained by dominant females during gestation. Analysis conducted on 12 females over 54 pregnancies* (23 Treated and 31 Control). Female identity entered as a random term.

**Explanatory terms**	**F**	**df**	***p***
Litter size	66.47	1	<0.001
Weight at conception (g)	17.38	1	<0.001
Rainfall during gestation (mm)	8.89	1	0.005
Treatment	5.62	1	0.022
Number of adults in group during gestation	1.0	1	0.32
Female age at conception (days)	0.12	1	0.734
Treatment order	0.77	1	0.39
Treatment year	0.0	1	0.97

**minimal model**	**effect size**	**s.e.**
Constant	234.6	12.75
Litter size	43.58	5.35
Weight at conception (g)	0.49	0.12
Rainfall during gestation (mm)	0.26	0.087
Treatment (Treated)	30.41	12.83

**Random Terms**	**Estimated variance**	**s.e.**
Female ID	125.8	78.3

*Sample size is less than complete experimental sample because it was not always possible to weigh dominant females within the target time windows.

Supplementary Table 9: LMM of variables influencing pup weight at first weighing. Analysis conducted on 215 pups (128 Treated, 87 Control) from 51 litters (22 Treated and 29 Control) born to 12 dominant females. Identity of mother and litter fitted as random terms.

**Explanatory terms**	**F**	**df**	***p***
Age at first weighing (days)	298.98	1	<0.001
Total rainfall between birth and first weighing (mm)	25.92	1	<0.001
Number of lactating females in group	24.59	1	<0.001
Litter size at emergence	15.70	1	<0.001
Treatment	6.46	1	0.021
Total rainfall during gestation (mm)	4.60	1	0.037
Number of adults in group at birth	3.02	1	0.09
Treatment order	1.33	1	0.27
Treatment year	0.83	1	0.37
Mother's weight at conception (g)	0.64	1	0.43
Pup sex	0.32	1	0.57
Mother's age at conception (days)	0.02	1	0.89

**minimal model**	**effect size**	**s.e.**
Constant	123.2	3.55
Age at first weighing (days)	3.94	0.23
Total rainfall between birth and first weighing (mm)	0.31	0.060
Number of lactating females in group	10.74	2.17
Litter size at emergence	-13.47	3.40
Treatment (Treated)	16.3	6.42
Total rainfall during gestation (mm)	0.084	0.039

**Random Terms**	**Estimated variance**	**s.e.**
Mother ID	20.5	62.7
Litter	434.8	113.1

Supplementary Table 10: LMM of variables influencing the number of allolactating females present during each breeding attempt. Analysis conducted on 59 breeding attempts (33 Control and 26 Treated) born to 12 dominant females. Identity of mother fitted as a random term.

**Explanatory terms**	**F**	**df**	***p***
Total rainfall during gestation (mm)	7.71	1	0.008
Treatment	7.59	1	0.008
Litter size at emergence	6.94	1	0.011
Mother's age (days)	5.49	1	0.025
Number of adults in group at birth	0.35	1	0.56
Treatment Year	0.31	1	0.58
Mother's weight at conception (g)	0.23	1	0.64
Treatment Order	0.25	1	0.62

**minimal model**	**effect size**	**s.e.**
Constant	2.84	0.28
Total rainfall during gestation (mm)	0.0046	0.0017
Treatment (Treated)	-0.96	0.35
Litter size at emergence	0.43	0.16
Mother's age (days)	0.0013	0.00053

**Random Terms**	**Estimated variance**	**s.e.**
Mother ID	0.407	0.312

Supplementary Table 11: LMM of variables influencing the rate at which subordinate females provisioned pups (in grams of food fed per minute of time observed). Analysis was conducted on 1050 focal watches, on 72 subordinate females. Response variable square root transformed. Individual identity and Mother identity fitted as random terms. Litter identity was not fitted as random term because pups from multiple litters were frequently present at the same time.

**Explanatory terms**	**F**	**df**	***p***
Number of pups under 90 days old foraging with group	23.59	1	<0.001
Treatment	5.80	1	0.016
Rainfall over previous 60 days (mm)	4.15	1	0.042
Number of adults in group	4.35	1	0.044
Individual weight (g)	4.68	1	0.032
Individual age (days)	0.35	1	0.56
Treatment year	1.15		0.30
Treatment order	1.48	1	0.23

**minimal model**	**effect size**	**s.e.**
Constant	0.049	0.0064
Number of pups under 90 days old foraging with group	0.0081	0.0017
Treatment	0.015	0.0064
Rainfall over previous 60 days (mm)	0.000082	0.000040
Number of adults in group	-0.0032	0.0015
Individual weight (g)	0.000089	0.000041

**Random Terms**	**Estimated variance**	**s.e.**
Mother ID	0.00017	0.00015
Individual ID	0.00026	0.00012

Supplementary Table 12: LMM of variables influencing the rate at which Dominant females provisioned pups (in grams of food fed per minute of time observed). Analysis was conducted on 1166 focal watches on 12 Dominant females. Identity of dominant female fitted as a random term. Litter identity was not fitted as random term because pups from multiple litters frequently present at the same time. Response variable square root transformed.

**Explanatory terms**	**F**	**df**	***p***
Number of pups under 90 days old foraging with group	38.71	1	<0.001
Individual weight (g)	25.08	1	<0.001
Number of adults in group	11.60	1	0.002
Reproductive status (non-breeding/pregnant)	5.41	1	0.021
Treatment Year	3.69	1	0.057
Treatment order	3.14	1	0.08
Age (days)	1.26	1	0.28
Treatment	0.48	1	0.49
Rainfall over previous 60 days (mm)	0.32	1	0.57

**minimal model**	**effect size**	**s.e.**
Constant	0.037	0.0044
Number of pups under 90 days old foraging with group	0.0081	0.0013
Individual weight (g)	0.00013	0.000026
Number of adults in group	-0.0041	0.0012
Reproductive status: Pregnant	0	
Non-breeding	0.011	0.0046

**Random Terms**	**Estimated variance**	**s.e.**
Dominant female ID	0.000081	0.000071

Supplementary Table 13: LMM of variables influencing the rate at which Dominant and Subordinate females provisioned pups in Control groups only (analysis conducted to investigate the effect of individual reproductive state on contributions to provisioning). Response variable square root transformed. Analysis was conducted on 1472 focal watches, on 12 Dominant and 72 subordinate females. Identity of Group and focal female fitted as a random terms. Litter identity was not fitted as random term because pups from multiple litters frequently present at the same time. Response variable square root transformed.

**Explanatory terms**	**F**	**df**	***p***
Number of pups under 90 days old foraging with group	34.09	1	<0.001
Number of adults in group	20.76	1	<0.001
Dominance status (Dominant or Subordinate)	8.58	1	0.006
Individual weight (g)	8.71	1	0.004
Reproductive status (non-breeding/pregnant)	6.02	1	0.014
Individual age (days)	4.22	1	0.054
Rainfall over previous 60 days (mm)	3.40	1	0.065
Treatment year	0.21	1	0.65
Treatment order	0.03	1	0.85

**minimal model**	**effect size**	**s.e.**
Constant	0.043	0.0040
Number of pups under 90 days old foraging with group	0.0073	0.0013
Number of adults in group	-0.0054	0.0012
Dominance status: Dominant	0	
Subordinate	0.043	0.0015
Individual weight (g)	0.000097	0.000033
Reproductive status: Pregnant	0	
Non-breeding	0.013	0.0054

**Random Terms**	**Estimated variance**	**s.e.**
Group ID	0.00026	0.00019
Individual ID	0.00021	0.00013

Supplementary Table 14: LMM of variables influencing pup weight between emergence and 95 days. Analysis conducted on 7620 morning weights, taken from 241 pups (141 males, 100 females) from 59 litters (26 Treated and 33 Control), born to 12 dominant females. Mother identity, Litter identity, pup identity, and pup identity*age were fitted as random terms.

**Explanatory terms**	**F**	**df**	***p***
Age (days)	3321.86	1	<0.001
Age^2	633.16	1	<0.001
Treatment*Age	4.35	1	0.03
Treatment* Age^2	30.66	1	<0.001
Total rainfall over previous 30 days (mm)	110.54	1	<0.001
Helper:pup ratio	79.12	1	<0.001
Number of lactating females during first 30 days of life	4.15	1	0.04
Treatment order	1.65	1	0.2
Treatment year	0.49	1	0.49
Sex	0.16	1	0.69

**minimal model**	**effect size**	**s.e.**
Constant	244.7	2.21
Age (days)	5.38	0.072
Age^2	-0.027	0.00056
Treatment*Age (Treated)	0.22	0.10
Treatment* Age^2 (Treated)	0.0043	0.00078
Total rainfall over previous 30 days (mm)	0.084	0.0080
Helper:pup ratio	4.39	0.49

Number of lactating females present during first 30 days of life	2.80	1.37

**Random Terms**	**Estimated variance**	**s.e.**
Mother ID	91.9	127.3
Litter ID	683.2	151.5
Individual ID	451.2	48.6
Individual ID * Age	1.4	0.2
